# An Improved Ecological Services Valuation Model in Land Use Project

**DOI:** 10.3390/ijerph16081474

**Published:** 2019-04-25

**Authors:** Zhichao Li, Tianqu Shao

**Affiliations:** 1 School of Political Science and Public Administration, East China University of Political Science and Law, Shanghai 201620, China; 2863@ecupl.edu.cn; 2 School of Public Administration, University of Electronic Science and Technology of China, Chengdu 611731, China

**Keywords:** environmental degradation, land use, true economic cost, Cobb–Douglas production function, differential equation

## Abstract

Natural ecosystems benefit human lives via providing fundamental life-support services and goods upon which human civilization depends. However, as nature provides those for free, many people believe that they are of little or no value and they exploit the land greedily and unreasonably, which makes serious ecological degradation. Concerning this issue, we present the ecological services valuation model (ESVM) to measure the cost of environmental degradation of land use cost, which is an evaluation model of environmental degradation cost. Environmental degradation cost refer to the cost of deterioration or compromise of natural environment through natural processes or human activities, which consists of opportunity cost and environmental damage cost. Land area is an important variable in the ESVM. Based on Osmotic system, we put forward the effective land area, which combines the scale factor and the impact of external environment. What is more, the Cobb–Douglas production function is modified to establish the model. Finally, we propose the calculation formula of the economic cost of land use projects. Analysis of effectiveness and sensitivity prove that ESVM was a relatively stable model.

## 1. Introduction

Ecosystem services [1] reveal a good man–nature relationship: The biosphere maintains a healthy environment for human life and provides many natural services via ecosystem services such as turning waste into food, pollinating plants, and converting carbon dioxide into oxygen. However, we usually destroy the environment intentionally or unintentionally, which leads to environmental degradation, limits ecosystem services functions, and endangers human life eventually.

The concept of ecosystem services accounting has emerged as a heated theme in international environmental circles in recent years. The Economics of Ecosystems and Biodiversity (TEEB) [2] is a global initiative focusing on “making nature’s values visible”. It aims to help decision-makers recognize many benefits provided by ecosystems and biodiversity, demonstrate their values in economic terms, and capture those values in decision-making [3].

In 2001, The Millennium Ecosystem Assessment (MEA) introduced a new framework for analyzing social-ecological systems that has had wide influence in the policy and scientific communities [4]. Chee et al. present a critical review on the neoclassical economic framework, tools used for economic valuation of ecosystem services, and the economic welfare approach to collective decision-making, from an ecological perspective [5]. Yang et al. establish a non-monetary accounting framework for ecosystem valuation model to propose new methods for biodiversity and climate regulation, and bridge the non-monetary and economic values [6].

Without considering the cost of ecosystem degradation, there will be potential risks. For example, when developing the land, people tend to ignore the economic value of ecosystem services, which brings threats to nature and human life [7]. Hence, it is unprecedentedly significant to consider the cost of ecosystem degradation in land use projects.

For a true and comprehensive valuation of land use projects, we need to create an ecological services valuation model (ESVM) that reflects the environmental costs we always ignore. Our model should be effective, feasible, and stable.

We intend to divide the environmental degradation cost into two parts; one is the opportunity cost, which means the loss of ecosystem services benefits, and the other is damage cost, which occurs in the process of unreasonable land use. Therefore, establishing an ESVM is crucial to other extended analysis.

Firstly, based on a functional-purpose perspective, we plan to divide ecosystem services into different subgroups and define each set of parameters and variables, then evaluate each subgroup separately. At the same time, we try to use a calculation model to unify the above evaluation methods. Then, we will collect the relevant official data of specific areas to build functions, which aim to analyze the damage costs of land use. We will combine the ESVM to further analyze the costs of environmental degradation.

Next, we also intend to take other factors into account, such as land prices, policy restrictions, infrastructure fees, and so on. We will standardize all the factors and comprehensively analyze the true economic costs of land use projects. Furthermore, we will add time parameters to the model to assess the impacts on ecosystem services when environment gets better or worse.

Moreover, for different scale land, we intend to refer to the theories such as benefit transfer and osmosis in biology, then analyze the effect of area factor. We will analyze the significance of our model for land use project planners and managers from the perspective of sustainable development and market regulations. Then, we will try to find a highly profitable way of land use and evaluate priorities among different ways. Furthermore, we will give an advisable suggestion by analyzing social responsibilities of market subjects.

Finally, in order to test reliability of our model, we will use official data to test the effectiveness, sensitivity, and robustness of the model.

## 2. Literature Review

### 2.1. History of Ecosystem Services Assessment

The assessment of ecosystem service value abroad can be traced back to 1925, when Drumarx first took the cost of wildlife recreation as the economic value of wildlife. Dafdon (1941) calculated the consumer surplus with the expense method taking the consumer surplus as the recreation value of the recreation area. It is he who put forward and applied the conditional value method. Clawson revised the travel cost method [8]. Knetch modified and improved the travel cost evaluation method [9]. In the same year, Davis studied the recreational value of Maine forests and first applied the conditional value method [10]. In 1972, the Japan forestry agency estimated the value of ecological functions provided by forests throughout Japan. Nordhau and Tobon proposed to revise the Gross National Produce (GNP) with the "economic welfare criterion", which aroused international attention to the estimation of environmental resources [11]. Many scholars have proposed various schemes to estimate the value of environmental resources. In 1991, the international scientific federation environment committee held a meeting to discuss how to carry out quantitative research on biodiversity, which promoted the development of biodiversity research and its value assessment methods. In 1993, the relevant United Nations agencies directed the publication of the interim edition of the handbook system of integrated environmental and economic accounting (SEEA), which provided a comprehensive summary of the previous studies on integrated environmental and economic accounting in various countries, as well as the overall thinking and framework of environmental and economic accounting, and some accounting methods of ecological value.

### 2.2. Introduction to Ecosystem Assessment Methods

One of the key challenges for ecosystem services research is to develop a comprehensive methodological approach in which biophysical, socio-cultural, and monetary value-domains can be explicitly considered and integrated into decision-making processes [12]. With the deepening of people’s understanding of the value of ecological services, the research methods are also expanding in depth, breadth, and direction. To enter widespread use, ecosystem service assessments need to be quantifiable, replicable, credible, flexible, and affordable. With recent growth in the field of ecosystem services, a variety of decision-support tools has emerged to support more systematic ecosystem services assessment [13]. In general, according to the research results of ecological economics, environmental economics, and resource economics, the main evaluation methods commonly used at present can be divided into three categories: Direct market method, including expense method, market value method, opportunity cost method, recovery and protection cost method, shadow engineering method, human capital method, benefit transfer, and a purely expert driven method [14], etc; alternative market method, including travel cost method and hedonic price method; and simulated market value method, including conditional value method. Despite broad recognition of the value of the goods and services provided by nature, existing tools for assessing and valuing ecosystem services often fall short of the needs and expectations of decision makers [15] and tools for identifying, assessing, modeling, and, in some cases, monetarily valuing ecosystem services have generally been lacking [13,16].The evaluation results may vary greatly due to the different evaluation methods. At present, there is no unified, standard, and perfect evaluation standard in the world.

### 2.3. Research of Ecosystem Services

In 1997, Costanza et al. were the first to estimate the system functional value of global biosphere ecosystem services [1]. The results, published in the journal Nature, have caused widespread concern and increased interest in the economic valuation of ecosystem services. At the same year, the book Ecosystem Service Function by Daily et al. systematically elaborated the content and evaluation method of ecosystem service function [17]. It analyzed nearly 20 examples of the evaluation of ecosystem service function in different regions, such as forests, wetlands, and coasts, which has high academic value. Since then, numerous studies have been published on the impact of land use change on ESV at global, national, and regional scales, including many catchment scale studies and multi-scale comparative studies. The study also focused on assessing individual ecosystem services values (ESVs) of forests, grassland, farmland, wetlands, and marine environments.

Urban green spaces provide numerous ecosystem services for city inhabitants, and the locally generated ecosystem services have a substantial impact on the quality-of-life in urban areas [18]. To be more specific, investing in ecological infrastructure in cities, and the ecological restoration and rehabilitation of ecosystems such as rivers, lakes, and woodlands occurring in urban areas, may not only be ecologically and socially desirable, but also, quite often, economically advantageous, even based on the most traditional economic approaches [19]. With the incresing urbanization, land use/cover change (LUCC) significantly impacts regional ecosystem services [20]. Meanwhile, there is neither a typical rural–urban gradient in terms of urban ecosystem service provisioning nor a uniform urban spatial pattern of service provisioning that can serve as a generic model for cities [21]. Therefore, it is urgent to put ecosystem services into consideration of land use planning including the city-specific context and perception but also the inhabitants’ preferences for cultural ES and existing substitutes [22].

Valuation of eco-services (in whatever units) is not the same as commodification or privatization [23]; in order to make better use of ecosystem services assessment, many scholars have discussed from different perspectives. Before assessing, it is necessary to define a conceptual framework and typology to describe, classify, and evaluate ecosystem functions, goods, and services in a clear and consistent manner [24]. Some scholars advocated consistently defined units of account to measure the contributions of nature to human welfare. They proposed a definition, rooted in economic principles, of final ecosystem service units. A goal of these units is comparability with the definition of conventional goods and services found in Gross Domestic Product (GDP) and the other national accounts [25]. The classification of ecosystem services should be based on interest characteristics and decision content [26]. Guerry et al. explored why ecosystem service information has yet to fundamentally change decision-making and suggested a path forward, and they advocated that working closely with leaders in government, business, and civil society to develop the knowledge, tools, and practices is necessary to integrate natural capital and ecosystem services into everyday decision-making [27]. Irvine et al. pointed that appreciation of the full value of ecosystem services requires recognition of values that are shared. They argued shared values thus do not necessarily exist a priori; they can be deliberated through formal and informal processes through which individuals can separate their own preferences from a broader metanarrative about what values ought to be shared [28].

Others have looked at the relationship between biodiversity and ecosystem services. Worm found positive relationships between diversity and ecosystem functions and services by using experimental and correlative approaches along trajectories of diversity loss and recovery. Their findings further suggested that the elimination of locally adapted populations and species not only impairs the ability of marine ecosystems to feed a growing human population, but also sabotages their stability and recovery potential in a rapidly changing marine environment [29]. Chan et al. found that biodiversity conservation protects substantial collateral flows of services. They presented an initial analytical framework for integrating biodiversity and found that, although there are important potential trade-offs between conservation for biodiversity and for ecosystem services, a systematic planning framework offers scope for identifying valuable synergies [30]. Gamfeldt et al. demonstrated that biodiversity has a positive impact on a variety of services and found a higher level of multiple ecosystem services in forests with more tree species. Therefore, they called on forest managers to consider multiple tree species [31]. 

Scholars have made specific assessments of the region. The ecological status of the semi-arid steppes in China is fragile. Wu et al. provided a new landscape ecological health (LEH) assessment approach, which can support landscape ecological restoration, ecological environmental protection, and urban planning of the semi-arid steppe mining cities [32]. Zhang et al. assessed the water quality status of the Qilihai wetlands to identify the pollution sources and potential improvements based on the ecological red line policy, to improve and protect the waters of the Qilihai wetlands. An indicator system was established to assess water quality status using single factor evaluation and a comprehensive evaluation method. Based on the research, they proposed restrictions on all polluting anthropogenic activities in the red line area and implementation of restoration projects to improve water quality [33]. Global warming and climate change increase the likelihood of weather-related natural disasters that threaten ecosystems and consequently affect the tourism industry, which thrives on the natural attributes of island regions. Chen constructed a random utility model using a choice experiment method (CEM) for the tourist resort on Orchid Island to study the factors enhancing tourist experiences [34].

## 3. Methods Research and Research Hypothesis

### 3.1. Method Research

Cobb–Douglas production function is a particular functional form of the production function, widely used to represent the technological relationship between the amounts of two or more inputs and the amount of output that can be produced by those inputs. The Cobb–Douglas was created and developed by Charles Cobb and Paul Douglas. They introduced the factor of technical resources to improve general production function [35].

The basic Cobb–Douglas production function model is as follows:

(1) $$Y=aK^{\alpha }L^{\beta }$$

In this formula, *Y* refers to production, *a* refers to technical level, *K* is the capital input, *L* is the labor input, and *α*, *β* is the output elasticity of *K* and *L*, respectively [36]. Ellis et al.’s production function analysis is a feasible method to evaluate the cost of ecological service function. It relies primarily on production or cost data, which are generally easier to obtain than the kinds of data needed to establish demand for ecosystem services [37].

Osmosis is the second theory we use in modeling; it is the spontaneous net movement of solvent molecules through a selectively permeable membrane into a region of higher solute concentration, in the direction that tends to equalize the solute concentrations on the two sides. It may also be used to describe a physical process in which any solvent moves across a selectively permeable membrane (permeable to the solvent, but not the solute) separating two solutions of different concentrations, which has been extensively studied by scientists in various disciplines of science and engineering.

### 3.2. Research Hypothesis

Due to lack of necessary data and limitation of our knowledge, we make the following assumptions to help us perform modeling:

(a) The benefits of ecosystem service can be transformed into corresponding economic benefits, while in the meantime, the damage to the environment can be quantified by economic cost. They can be analyzed by the production function.
(b) True economic cost of land use projects include two parts: One is the environmental degradation cost related to natural environment, the other is the social cost related to human input.
(c) The data used in this paper are reliable.

## 4. Establishment of the Ecological Services Valuation Model (ESVM)

### 4.1. Ecological Services Valuation Model (ESVM)

This model is required to measure the ecological services benefit of land. At the first step, we need to define the effective area of land, We use *S* to denote the effective area of land.

As the ecosystem is open and interactive, we take into account external factors. To simplify the model, we assume there is a rigorous boundary to distinguish the ecosystem and external environment. External environment will limit the ecological system functions, which reveals the essential difference of the two conceptions. That is, if a part of the external environment has no bad influence on the ecosystem, then it will belong to the ecosystem [38].

Due to the similarity to osmosis, we can regard the boundary as semipermeable membrane, then the external effects on the ecosystem, and the process of environmental impacts on ecosystems is analogous to solvent molecules passing through semi-permeable membranes.

Base on the analysis above, we can define that the effective area of land is the area where the land can play its service function effectively [39].

Subsequently, we use the two-dimensional normal distribution to simulate the influence of external environment on the certain area. The probability density function of the two-dimensional normal distribution is as follows.

(2) $$f\left(x,y\right)=\left(2\pi \sigma_{1}\sigma_{2}{)}^{-1}exp[-\frac{1}{2}\left(\frac{{\left(x-\mu_{1}\right)}^{2}}{\sigma_{1}^{2}}+\frac{{\left(y-\mu_{2}\right)}^{2}}{\sigma_{2}^{2}}\right)\right]$$

In the calculation process, we specify *μ*_1_ = *μ*_2_ = 0, which has no effect on our calculation results and graphics. *σ*_1_, *σ*_2_ are proportional to the width *a* and length *b* of the land area, respectively. Considering the simplicity and rationality of the model, we specify:

(3) $$\{\begin{array}{c} \sigma_{1}=3ln(a)\\ \sigma_{2}=3ln(b) \end{array}$$

Next, we define *S_l_* is the land area limited by the external environment, which is π times the integral of a two-dimensional normal distribution over this region.

**Figure 1:**
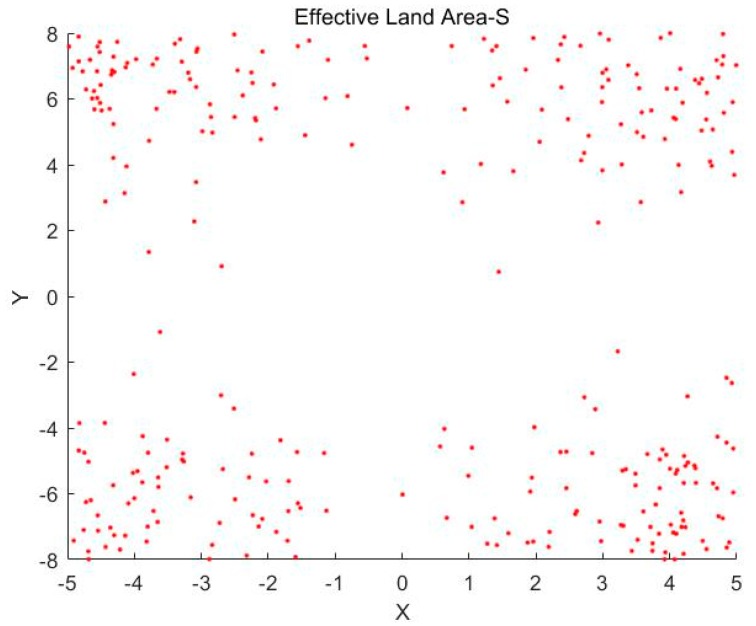
The impact of external environment on land.

Then, we can get the random points of two-dimensional normal distribution, and centrally symmetrical to four corners to represent the constraints of the external environment on the ecosystem, as shown in Figure 1.

From Figure 1 above, we can find that different areas of land have been affected to varying degrees. For the edge of land, especially the corner of the land, it is directly affected by the outside world, so the land that it is unstable and weak in reality. For the land around the central part, it is less affected by the outside world, so the reality shows that it is relatively stable and capable.

The formula to calculate the effective land area *S* is as follows:

(4) $$S=ab-S_{l}.$$

Table 1 shows examples of calculations for three different scales of land.

From the table, we can conclude that along with the growth of scale, the effective land area proportion is increasing and the land ability for ecosystem functions is enhanced; that is, ecosystems will have stronger resistance with the increase of scale, which proves that our model is in line with objective reality.

Then, we apply the Cobb–Douglas production function to the ESVM, and establish the Cobb–Douglas production function equation corresponding to eight functions of ecosystem services.

(5) $$B_{i}=a_{i}S^{\alpha_{i}}L_{i}{}^{\beta_{i}},i=1,2,...,8$$

Variables have the following meanings in Table 2: Eight values of *i* represent eight functions of ecological services.

*B_i_* is the corresponding ecosystem services function benefit, *a_i_* is the corresponding ecosystem service level, *S* is the effective land area, *Li* is the specific parameter of different functions. Table 3 shows their meanings.

Explanation of L7: The ability to purify the environment. Considering absorption and transformation of green plants contribute most in the process, here we take the sum of the surface area of green plant leaves as the parameter. *αi* and *βi* are the elastic coefficients of *S* and *L*. Since *S* already includes the impact of scale on the benefits of ecological services, so *αi* + *βi* = 1, which means that there is no scale benefit in ecological services.

Considering that it is impossible for a specific ecosystem to have the above eight functions at the same time, an ecosystem will be equipped with different service functions. Because humans can choose to plant crops uniformly on a specific land or even no planting, such land has functions 1, fuction 5 and function 6, or uphold the principle of maintaining biodiversity and enriching plant varieties, has functions 2 and fuction 8. But they both have functions 3, fuction 4 and function 7.

Therefore, the formula to calculate the functional benefits of ecosystem services is as follows:

(6) $$B=\overset{8}{\underset{i=1}{\sum }}k_{i}B_{i}$$

where *k_i_* (*i* = 1–8) is the function coefficient of ecosystem services; there are two values: 0 and 1. *k_i_* = 0 means that the land does not have corresponding functions, while *k_i_* = 1 means that the land has corresponding functions.

### 4.2. The Cost Calculation of Environmental Degradation

In the process of land use development, human beings not only make the land lose its own ecosystem services capability, but also do great damage to the ecosystem. Therefore, it is necessary to take the cost of environmental degradation into account of land use cost. The cost of environmental degradation includes two parts: The opportunity cost of human land use projects and the environment damage cost of the projects. The former is equal to the benefits of the ecosystem services, which is expressed as opportunity cost *C_oppo_*. The latter is the damage to the natural environment caused by the project itself, expressed as *C_loss_*. Opportunity cost *C_oppo_* and eco-service benefit *B* are the same in value. So, we can get:

(7) $$C_{oppo}=B$$

Environment damage cost mainly means the negative effect to natural capital, which is presented as air pollution, soil pollution, climate change, biodiversity damage, and hazardous waste treatment [40].

Since human behaviors cannot cause great climate change in a short period of time, we leave out the factor. Since landfill is the common treatment method for hazardous waste such as chemical experimental waste and some industrial waste, and the impacts of hazardous waste will finally be manifested as soil pollution, we leave out it as well. We also removed the value of biodiversity because it is hard to quantify and it is unrepresentative to take the cost of a specific event as the biodiversity value. Considering that ecological environment is an interconnected system and the value of biodiversity has been integrated into all aspects of the environment, we remove the value of biodiversity to avoid double calculation.

Since the varied damage to the environment is difficult to quantify, we convert the environment damage cost into the cost of human pollution prevention. For example, we can convert the water pollution damage cost into human water pollution prevention cost. In addition, we also take the self-healing ability of the natural environment into account. Subjected to these constraints, we can obtain:

(8) $$\{\begin{array}{l} C_{water}=c_{1}M_{water}\\ C_{air}=c_{2}M_{air}\\ C_{soil}=c_{3}S_{soil}\\ C_{loss}=C_{water}+C_{air}+C_{soil}-B_{heal}\\ C_{loss}\geq 0 \end{array}$$

where *C_water_*, *C_air_*, and *C_soil_* mean the environment damage cost of water pollution, air pollution, and soil pollution, respectively. *B_heal_* means the benefits of self-healing ability of the natural environment. *M_water_* and *M_air_* mean the quality of polluted water and polluted air, respectively. *S_soil_* is the area of polluted soil. *C*_1_, *C*_2_, and *C*_3_ are human compensation coefficients, which mean the per-unit cost of human pollutiond prevention (*C*_1_ = 103 dollar/t [13], *C*_2_ = 64.96 dollar/t [14], *C*_3_ = 13.07 dollar/m^2^ [15]).

**Figure 2:**
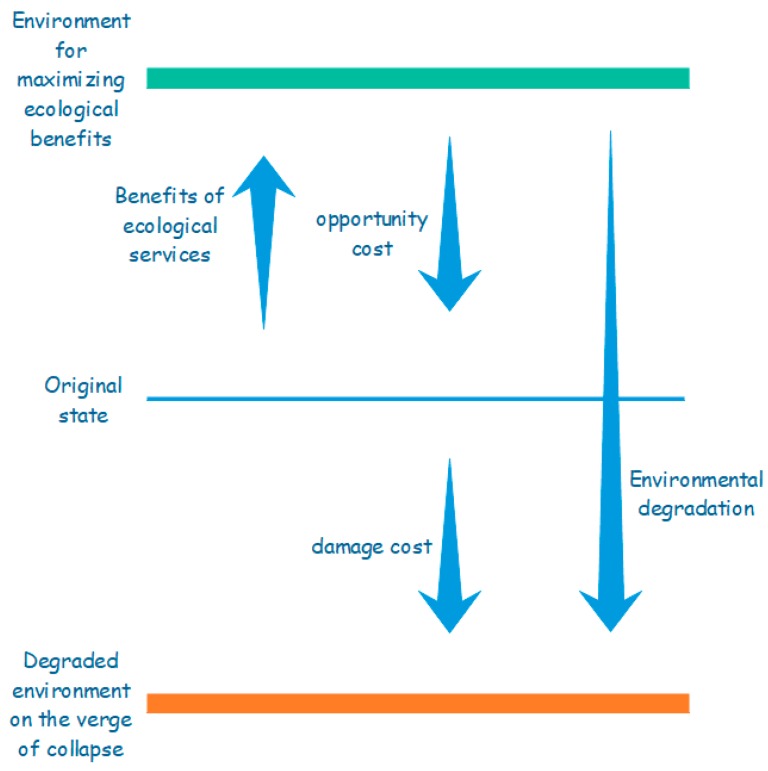
The concept of the environmental degradation cost.

On the basis of the Equation (8), we further obtain the environmental degradation cost (*C_ED_*). Figure 2 shows the concept of the environmental degradation cost, which is the sum of opportunity cost (*C_oppo_*) and damage cost (*C_loss_*). The formula is characterized as:

(9) $$C_{ED}=C_{oppo}+C_{loss}$$

### 4.3. Modified Model with Time Parameter

**Figure 3:**
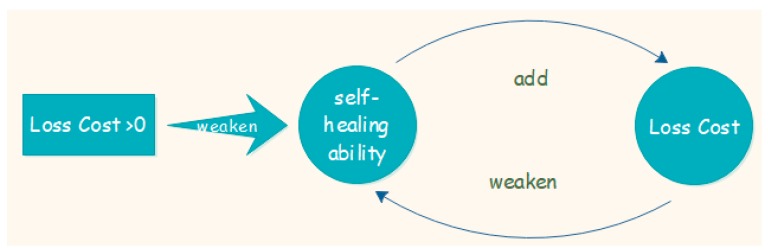
Vicious circle of environmental degradation.

As we mentioned the self-healing ability of natural environment in above analysis, we explain its calculation method here. Natural environment can heal itself over time, however, the ability performs obviously when human beings destroy the environment. The ability is also the object of the vicious circle of environmental degradation, which means the degradation of the environment at the current stage will weaken the self-healing ability of the next stage. So, the environment at the next stage will degenerate into a more fragile condition (see Figure 3).

Therefore, time affects the environment damage cost by affecting the self-healing ability of the natural environment, thus affecting the overall true economic cost of the land use projects. Finally, we further analyze the self-healing ability benefit calculation method based on different function. Then, we introduce:

(10) $$\{\begin{array}{l} B_{heal}\left(0\right)=b\\ \frac{dB_{heal}\left(t\right)}{dt}=rC_{loss}t+r_{0}\\ 0\leq B_{heal}\leq b \end{array}$$

where, *B_heal_* (t) is the self-healing ability of natural environment at *t* moment, and *b* is the initial value of *B_heal_*.

### 4.4. The True Economic Cost of Land Use Projects

It is unscientific that the original economic cost only includes the social cost paid by human beings in land use projects, and the social cost is the human input of purchasing production materials and labor. Environmental input of projects may seem inconsequential to the total costs, however that is not comprehensive. Combined with the relevant factors we considered before, we can identify that the true economic cost of land use projects (*C_true_*) should include the social cost (*C_s_*) and the environmental degradation cost (*C_RD_*), i.e.,

(11) $$C_{true}=C_{S}+C_{RD}$$

## 5. Application of the ESVM

### 5.1. Parameter Estimation Based on Valid Data

On the basis of crop quantity, forest area, photosynthetic efficiency of plants, precipitation, increasing agricultural production by soil function, number of pollinators, the ability to purify the environment, and coverage of cultural and entertainment facilities, we estimated the value of each parameter in modified Cobb–Douglas production function model (5). According to the above data, we can get Table 4.

### 5.2. Cost–Benefit Analysis of Different Scale Projects

Considering the limited data of the full cost of a specific land use project, we simulate two different kinds of land use projects: Small-scale agricultural land A and large-scale industrial land B. We give the final results of ecological service benefit B and damage cost *C_loss_* based on Equations (6) and (8). The cost data of land use projects calculated by the modified Cobb–Douglas production function model are as in Table 5.

**Figure 4:**
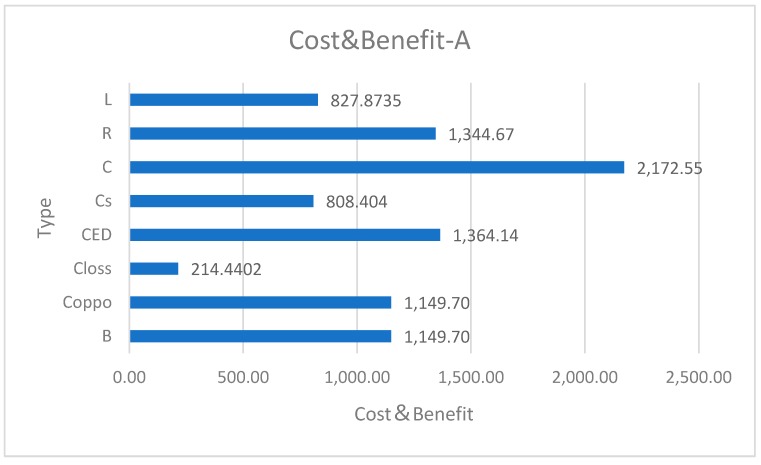
Cost and benefit of Project A.

**Figure 5:**
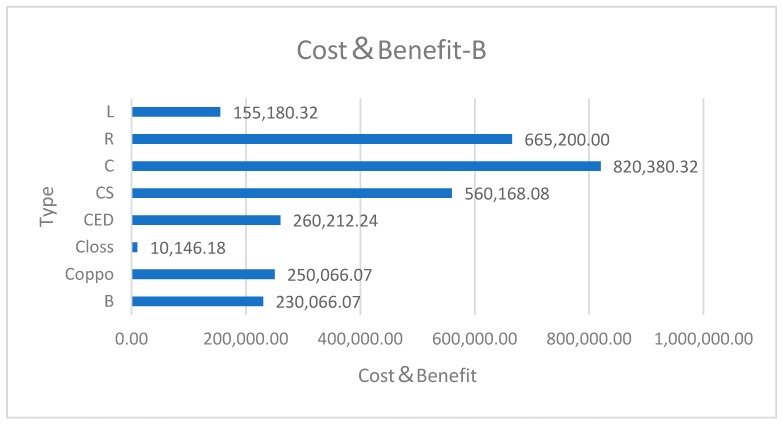
Cost and benefit of Project B.

The data *n* in Table 5 are shown as follows by bar chart (Figure 4 and Figure 5).

Data show that both projects have positive profits without considering ecological services factors. But after considering ecological services, which means that humans need to mitigate the negative results of environmental degradation, both projects have negative profits, i.e., losses. Even if the land type has changed from agricultural land to industrial land, using for industry is more profitable than using for agriculture. But the change still has not offset the impact of environmental degradation. At the cost of environmental degradation, people seem to have made profits, but in fact they are losing a lot such as water storage benefits of wetlands.

### 5.3. True Economic Cost Changes with Time Factor

**Figure 6:**
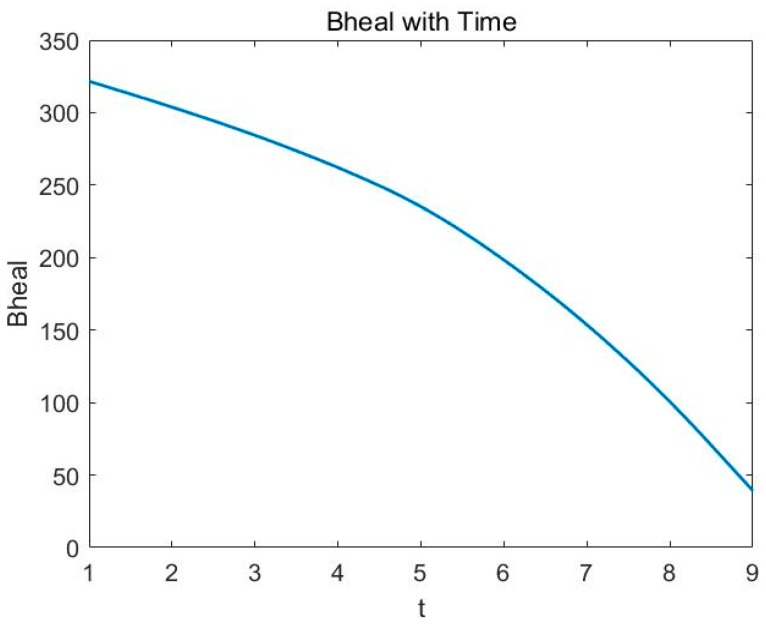
Self-healing ability changes with time factor.

Based on historical data from the national bureau of statistics in China, we convert the parameter estimation problem of differential Equation (10) into multi-variable linear regression problem and get the related parameters, where r is −0.187 and r_0_ is 23.028. After considering the time, the self-healing ability of the natural environment is directly affected by the time and the ability changes as shown in Figure 6.

In Figure 6, the horizontal axis is time and the vertical axis is self-healing ability benefit. As time goes on, the decline in self-healing ability will be faster and faster.

**Figure 7:**
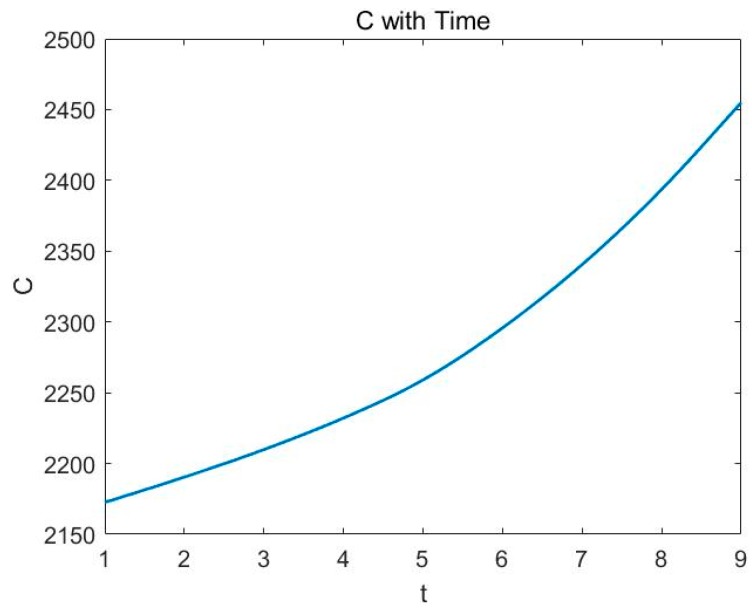
The true economic cost changes with time factor.

Therefore, the true economic cost of the land use projects will also be affected eventually. These results are shown in Figure 7.

In Figure 7, the horizontal axis is time and the vertical axis is the true economic cost of land use projects. As time goes on, the true economic cost will increase faster and faster.

## 6. Sensitivity Analysis

The state or output variation of the model always depends on the changes of system parameters or surrounding conditions. Sensitivity analysis aims at studying the sensitivity of such dependencies. Adjust the data slightly and recalculate them back to the model, then observe the changes in the results, which are considered the common method of sensitivity analysis [41].

**Figure 8:**
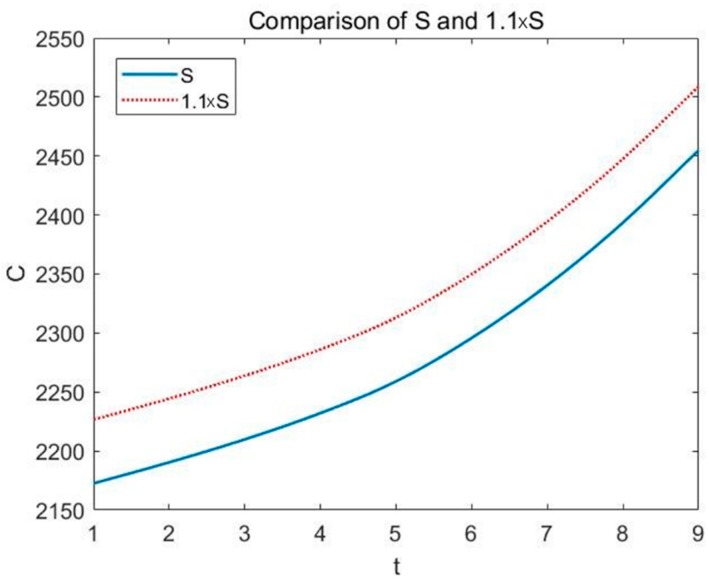
Change of *C* when *S* enlarges by 1.1 times.

**Figure 9:**
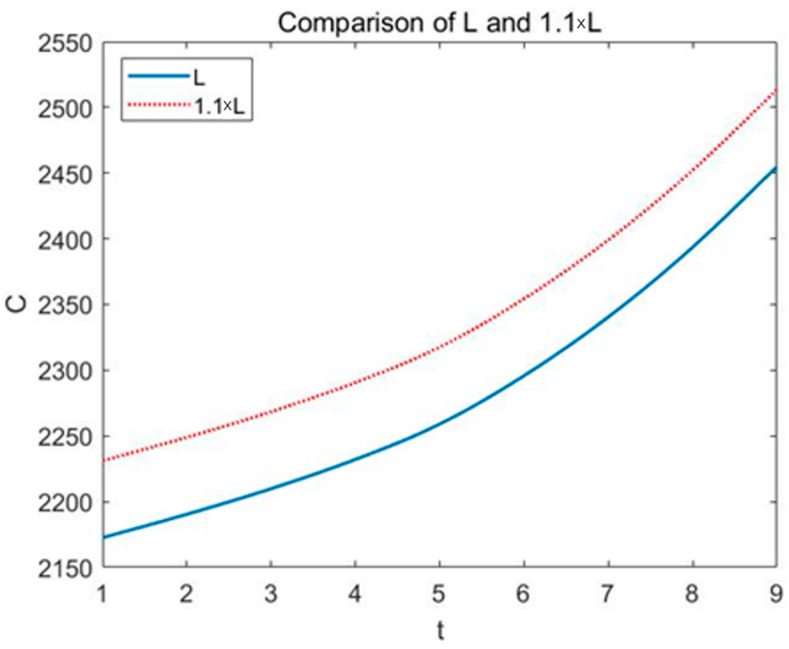
Change of *C* when *Li* enlarges by 1.1 times.

The model proposed in this paper involves several indicators, but most of the indicators are based on the effective area of land *S* and the specific index *Li* of different ecological service functions. Therefore, we added small disturbances to *S* and *Li*. We increased the original data to 1.1 times to see the changes on true economic cost of land use projects. The result is shown in the following figures (see Figure 8 and Figure 9).

In the figures, the horizontal axis is time and the vertical axis is true economic cost. It can be seen clearly that the true economic cost *C* increases with the growth of *S* and *Li*. By calculating, we get the standard deviations of the two curves are 51.2502 and 55.5490, respectively. That is, when *S* changes by 10%, *C* changes by 2.36%, and when *Li* changes by 10%, *C* changes by 2.56%. Sensitivity analysis results show that the model does not depend on the two basic indicators of effective land area *S* and specific parameters *Li* of different ecological service functions, and the model performs well.

## 7. Discussion and Conclusions

### 7.1. Evaluation of Model

Our model has some strength. Firstly, Cobb–Douglas production function model [35] is used in our model, which effectively solves the difficult problem of quantifying the functional benefits of ecosystem services. Subsequently, because the ecosystem is open, we take into account the external effects on ecosystem functions. We introduce the effective area of land to analyze the effect. What is more, our model covers a comprehensive range of evaluation, including several main types of costs in land use projects. Moreover, we take into account the vicious circle in the process of environmental degradation. Eventually, the model effectiveness results show that the proposed model is effective and stable. In the meantime, by comparison and conclusion, our model also has several weaknesses. Initially, the data needed by the model are relatively uncommon and there are too much data to collect. So, we select several major ones and use computers to simulate related data. Thus, the representativeness of data needs to be further strengthened. Two-dimensional normal distribution may be not the best choice to simulate the osmosis process. In other words, there may be a more adaptable model to solve the problem. What is more, the mixed ecosystems and land use projects are not considered.

### 7.2. Conclusions

In this paper, we create the ESVM for ecosystem services and expressions for the true economic cost and the environmental degradation cost. Based on the above formulas, we analyze cost–benefit of different scale land projects. Then, we find that a seemingly profitable land use project may lose its natural capital after adding the cost of environmental degradation, and may lose more over time. In order to test the robustness of our model, we analyze the sensitivity and effectiveness of the ESVM based on real data. When the important parameters in the model increase by 10%, the true economic cost changes only about 2%. Therefore, the model we came up with has strong robustness and effectiveness.

### 7.3. Model Improvement

Recent years have witnessed an increasing number of people thinking highly of environment protection, gradually. In 2012, the United Nations put forward the goal of sustainable development to call on individuals to live in harmony with nature. Ecosystems are destroyed by human beings arbitrarily in that there is no charge for products and services provided by nature, which has caused the destruction of ecosystems and will eventually endanger our lives.

In order to solve the problem, we innovatively create a valuation model to evaluate the true economic cost of land use projects; in the process, we build a related model to evaluate costs of ecosystem services and environmental degradation.

However, as discussed in the weaknesses of the models, the limited amount of data may influence the accuracy of the results. So, the model needs to be further modified; for example, the model to calculate the effective area of land should be perfected.

Therefore, in the future, we will improve our model to make it more comprehensive and definitive. Most importantly, we desire that the model contribute a lot to optimize the man–nature relationship and achieve the goal of sustainable development.

## Figures and Tables

**Table 1:** Examples of three different scales of land.

Land Scale	Restricted Land Area (km^2^)	Effective Land Area (km^2^)	Total Land Area (km^2^)	Effective Proportion
3 × 4	1.632	10.368	12	86.40%
8 × 10	1.459	78.541	80	98.17%
10 × 16	1.758	158.242	160	98.90%

**Table 2:** Meanings of eight values of *i*.

1	Production of the organic matters and natural resources	5	Soil retention and formation
2	The maintenance of biodiversity	6	Habitat provision and pollination
3	Climate regulation	7	Purification of environment
4	Disturbance prevention	8	Cultural services

**Table 3:** Meanings of *Li*.

L_1_	Crop quantity	L_5_	Increased agricultural production by soil function
L_2_	Forest area	L_6_	Number of pollinators
L_3_	Photosynthetic efficiency of plants	L_7_	the sum of the surface area of green plant leaves
L_4_	Precipitation	L_8_	Coverageof cultural and entertainment facilities

**Table 4:** Parameters in modified Cobb–Douglas production function model.

		*a*	*α*	*β*
		(Technical Level)	(Output Elasticity of *S*)	(Output Elasticity of *Li*)
1		27.33089	0.400749	0.599251
2		162.2141	0.133845	0.866155
3		95.52251	0.605917	0.394083
4		2.895428	0.291515	0.708485
5		0.917527	0.503798	0.496202
6		1.207112	0.827084	0.172916
7		14.22006	0.157246	0.842754
8		6.723872	0.847115	0.152885

*S* is the effective land area and *Li* is the specific parameter of different functions.

**Table 5:** Parameters in modified Cobb–Douglas production function model.

Parameter data	Small-scale land A	Large-scale land B
Land type	Agricultural land	Industrial land
Land specifications (a × b)	3 × 4	8 × 10
Effective land area S	10.368	78.541
Ecological services B	1149.7013	250,066.0650
Opportunity cost Coppo	1149.7013	250,066.0650
Environment damage cost Closs	214.4402	10,146.1769
Environmental degradation cost CED	1364.1415	260,212.2419
Social cost Cs	808.4040	560,168.08
True economic cost C	2172.5455	820,380.3219
Income R	1344.6720	665,200.0000
Real profit P	−827.8735	−155,180.3219
